# Medical Inspection of Schools
*"Medical Inspection of Schools." By A. H. Hogarth. (Mr. Henry Frowde and Messrs. Hodder & Stoughton.)


**Published:** 1910-08-20

**Authors:** 


### Public Health and Hygiene.

### MEDICAL INSPECTION OF SCHOOLS.*

The medical inspection of school children has been claimed as a branch of medicine—in some particular sense a speciality requiring a peculiar knowledge and even a rare and somewhat extraordinary personality. Dr. Hogarth sets out the desirable attributes of the school medical officer as follows: "He should be a man of wide experience, of large sympathies, of many interests, and endowed with abundant insight into human nature. Such personal qualities are likely to prove of greater value than the highest professional attainments. The school doctor should have a special temperament and should possess some of the characteristics more commonly found in women than in men, such as the power of attraction, sympathy, and a natural love for children. . . The school doctor's professional qualifications are also of importance. Generally speaking, he should begin to fill his first school appointment between the ages of 30 and 35; but for routine work older men may very reasonably be chosen if they have been engaged in the practice of medicine. Over and above the ordinary training of a medical student a would-be school doctor should hold an appointment for six months or a year as house physician or house surgeon in a general hospital. He should also devote some time after qualification to making himself specially acquainted with the practical methods and routine of the special departments concerned with diseases of the eye, ear, nose, throat, and skin, and also with a special study of orthopædics and of electrical treatment for infantile paralysis. Then he should spend at least six months in the wards and out-patient department of a children's hospital, where he would study the normal and pathological growth and development of the child, and another six months either in a fever hospital or in an asylum for mental diseases. That is to say, for two or three years after obtaining a qualification the would-be school doctor devotes his time to attaining some degree of proficiency in all the branches of his profession." This by no means exhausts the catalogue of requirements, personal and professional, which, according to Dr. Hogarth, will justify a medical practitioner in looking for "an appointment as local school doctor in charge of a small group of schools in his neighbourhood."

It is at least a relief to find that this Admirable Crichton transcends the mundane limitations of the mere practitioner of physic and "plays the part, as it were, of a professional pedagogue, not making the children introspective and neurotic, but in the case of the elder children impressing upon them the object of his visit and the need of their co-operation and good sense."

Since in Dr. Hogarth's estimation the school doctor is to masquerade as a professional pedagogue, and to be employed less in the diagnosis of disease than in the psychological gymnastics of the lightning interpretation of facial expression, we are not surprised to learn that "so far as systematic medical inspection is concerned, sanitation and the prevention of acute infectious disease are merely secondary considerations."

It is the vogue of the newer school hygienists to pooh-pooh the importance of infectious diseases and to relegate them with "drains," by which is connoted "sanitation," to a somewhat obsolete functionary, the medical officer of health. Whatever relative importance may in the future attach to infectious disease in schools, it must be remembered that school hygiene took its origin in the attempts made to control these diseases. We recognise that school hygiene comprises the application of medical knowledge in the fullest sense to educational problems, and we desire to say no word in derogation of this important expansion of medical work, but we fail to see that any good purpose is served by the attempts to belittle the work of infectious disease prevention among public school children.

To close the eyes to the part played by the schools in the spread of these diseases is not to advance the cause of school hygiene. Dr. Hogarth says: "In consequence of the notable decrease of cases of infectious disease during the holidays—to which Sir Shirley Murphy first drew attention in 1896—it has been generally assumed that the majority of cases are spread by means of contact in school."

When we are informed that "Dr. Kerr has shown that, owing to the exodus of children from the towns during the holidays, an alteration in the distribution of population takes place which to some extent may account for the decrease in the number of scarlet fever cases," we feel that Sir Shirley Murphy's valuable contribution as to the part played by schools in the spread of this disease is receiving less than fair treatment.

The remarkable decline in August of scarlet fever and diphtheria notifications has been shown repeatedly so to accord in its relations as to incubating periods, age incidence, and place distribution, with school closure that no doubt remains in the minds of experienced observers as to the influence of school attendance upon the spread of these diseases.

Dr. Hogarth's book abounds with polemical discussions wholly unsuited to textbook treatment. For better or worse school hygiene has been recognised a branch of preventive medicine, and, with rare exceptions, identified or co-ordinated with the public health service. The process has not been one of fusion, for school hygiene has grown out of public health administration, and from the nature of the case must ever remain an outgrowth co-ordinate with others of lesser or greater importance. So far the literature of this branch of the work is not encouraging or instructive reading. It should be both, for the field is promising; but it seems necessary to remind the school medical officer that he is no more a pedagogue than the medical officer of health is an inspector of nuisances.

* "Medical Inspection of Schools." By A. H. Hogarth. (Mr. Henry Frowde and Messrs. Hodder & Stoughton.)

